# Towards a Multi-Enzyme Capacitive Field-Effect Biosensor by Comparative Study of Drop-Coating and Nano-Spotting Technique

**DOI:** 10.3390/s20174924

**Published:** 2020-08-31

**Authors:** Denise Molinnus, Stefan Beging, Carsten Lowis, Michael J. Schöning

**Affiliations:** 1 Institute of Nano- and Biotechnologies (INB), FH Aachen, Campus Jülich, 52428 Jülich, Germany; molinnus@fh-aachen.de (D.M.); beging@fh-aachen.de (S.B.); carsten.lowis@alumni.fh-aachen.de (C.L.); 2 Institute of Biological Information Processing (IBI-3), Research Center Jülich, 52425 Jülich, Germany

**Keywords:** multi-enzyme immobilization, nano-spotting, field-effect EIS biosensor, penicillinase, urease, glucose oxidase

## Abstract

Multi-enzyme immobilization onto a capacitive field-effect biosensor by nano-spotting technique is presented. The nano-spotting technique allows to immobilize different enzymes simultaneously on the sensor surface with high spatial resolution without additional photolithographical patterning. The amount of applied enzymatic cocktail on the sensor surface can be tailored. Capacitive electrolyte-insulator-semiconductor (EIS) field-effect sensors with Ta_2_O_5_ as pH-sensitive transducer layer have been chosen to immobilize the three different (pL droplets) enzymes penicillinase, urease, and glucose oxidase. Nano-spotting immobilization is compared to conventional drop-coating method by defining different geometrical layouts on the sensor surface (fully, half-, and quarter-spotted). The drop diameter is varying between 84 µm and 102 µm, depending on the number of applied drops (1 to 4) per spot. For multi-analyte detection, penicillinase and urease are simultaneously nano-spotted on the EIS sensor. Sensor characterization was performed by C/V (capacitance/voltage) and ConCap (constant capacitance) measurements. Average penicillin, glucose, and urea sensitivities for the spotted enzymes were 81.7 mV/dec, 40.5 mV/dec, and 68.9 mV/dec, respectively.

## 1. Introduction

These days, progress in nanoscience is dealing with the miniaturization of biosensors together with multi-analyte detection provided by ideally one single chip. Such multi-analyte detection methods are interesting for a wide range of on-line applications, where the quantitative and rapid evidence of several analytes in parallel is necessary [1,2,3,4]. Biosensors with immobilized enzymes represent one class of such inexpensive and small analytical devices; they are by far the most commonly used signaling method for the detection of different analytes in medicine, food quality control, environmental monitoring, and research [5,6,7,8,9,10,11]. The immobilization of costly biomolecules (e.g., enzymes, DNA, and antibodies) onto the sensor surface often demands their reuse for at least several times. Apart from being cost-effective, the ideal immobilization technique has to fulfill numerous characteristics such as stability, regenerability, inertness, and no influence on the enzymes’ activity [12,13,14]. Several enzyme-immobilization procedures can be found in literature, like cross-linking, encapsulation, and drop-, spin-, and dip-coating [15]. However, these methods are commonly time-consuming and immobilization of small amounts (nanoliters) of enzyme solution is quite challenging with regard to a localized and spatial-resolved enzyme immobilization. Furthermore, the co-immobilization of several enzymes with a particular spatial resolution onto one sensor surface by using conventional immobilization techniques is difficult to accomplish. In the past, different methods for immobilization of small solution amounts onto sensor surfaces have already been developed by using an e.g., nano-dispenser to spread solution in the sub-micron scale onto a target [16,17,18,19] or a droplet spotter to deposit pico- to femtoliter droplets with a high density on chemicals on a surface [20,21]. In contrast, there is only little experience in nano-spotting enzyme immobilization for the development of miniaturized biosensors, especially with regard to co-immobilization of several enzymes onto the same chip surface, addressing defined areas to develop an enzymatic multi-analyte biosensor. Recently, a nano-spotter dispenser was suggested for the spatially resolved immobilization of the enzyme penicillinase [22]. In that study, first attempts were made to investigate the sensor characteristics of a fully and half-spotted capacitive field-effect sensor towards different penicillin concentrations. However, only one enzyme was immobilized in this “proof-of-concept” experiment. Therefore, in the present work, a fundamental research was done with capacitive field-effect sensors varying the selected areas of enzyme immobilization by nano-spotting technique, i.e., full-, half-, and quarter-spotted surface area of sensor chip with enzyme droplets, and investigating three different enzymes (penicillinase, urease, and glucose oxidase) as well as their co-immobilization (urease and penicillinase). The co-immobilization of two enzymes mimics the first step towards a multi-analyte capacitive field-effect biosensor utilizing one single sensor chip.

## 2. Materials and Methods

### 2.1. Reagents

Penicillinase (E.C. 3.5.2.6, from *Bacillus cereus*, specific activity: 1500–3000 U/mg), β-d-glucose oxidase (EC 1.1.3.4., from *Aspergillus niger*, specific activity: 145,200 U/g), urease (E.C. 3.5.1.5, from *Canavalia ensiformis*, Jack bean, specific activity: 76,440 U/g), glucose monohydrate and penicillin G were purchased from Sigma-Aldrich (Germany). Urea was bought from Fluka Analytical (Germany). Triethanolamine hydrochloride buffer (TEA, C_6_H_15_NO_3_Cl, Sigma-Aldrich, Germany) was prepared as stock solution (0.3 M, pH 6). For the preparation of the polymix multi-component buffer solution (PBS, 25 mM buffer capacity), following chemicals were dissolved in deionized water: 3.029 g/L tris-(hydroxymethyl)aminoethane (C_6_H_11_NO_3_, Sigma-Aldrich, Germany), 3.402 g/L potassium dihydrogen phosphate (KH_2_PO_4_, Merck, Germany), 5.254 g/L citric acid-1-hydrat (C_6_H_8_O_7_ · H_2_O_,_ Merck, Germany), 5.031 g/L di-sodium tetraborat (Na_2_B_4_O_7_^.^ H_2_O_,_ Merck, Germany), and 1.864 g/L potassium chloride (KCl, Sigma-Aldrich, Germany). This stock solution was diluted with deionized water to get a 0.25 mM PBS. Additionally, as ionic strength adjuster, 7.456 g/L KCl powder was dissolved into the diluted PBS resulting in a 100 mM concentration.

### 2.2. Preparation of Sensor Structure

**Figure 1:**
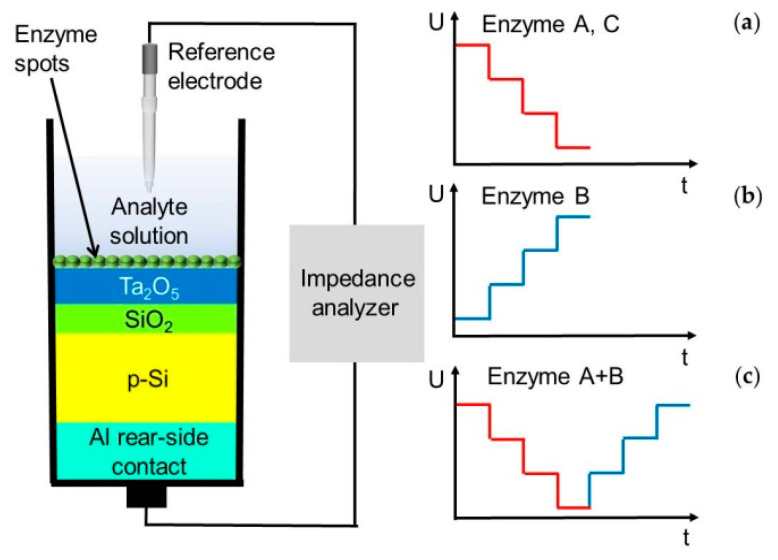
Schematic measurement set-up of modified capacitive electrolyte-insulator-semiconductor (EIS) sensor by a fully spotted enzyme layer on top (left); expected constant capacitance (ConCap) curve depending on the applied enzyme A (penicillinase) (**a**), B (urease) (**b**), C (glucose oxidase) (**a**), or A and B together (**c**) on the same chip (right).

The capacitive electrolyte-insulator-semiconductor (EIS) sensor with Ta_2_O_5_ as pH-sensitive material was fabricated by means of thin-film technology under cleanroom conditions. A detail description can be found in [23,24]. In short, a p-doped silicon substrate (thickness: ~400 µm, specific resistance ρ = 5–10 Ω cm) was used to form a 30 nm SiO_2_ insulating layer by thermal oxidation under oxygen atmosphere at 1000 °C for 30 min. Afterwards, a 30 nm Ta layer was deposited by electron-beam evaporation, followed by thermal oxidation at 520 °C for 45 min to Ta_2_O_5_ with a thickness of approximately 60 nm as excellent pH-sensitive transducer material [25,26]. Finally, as rear-side contact 300 nm Al was deposited by electron-beam evaporation, which is annealed afterwards. The final wafer was diced by a diamond saw to 1 × 1 cm² sensor chips. Figure 1 (left) shows the cross-sectional schematic of the different layers.

Two different immobilization strategies were investigated for comparison: (i) conventional drop-coating technique by applying an adjustable pipette (Eppendorf Research^®^ plus, Germany) to deposit the enzyme solution onto the sensor surface, and (ii) nano-spotting technique by utilizing the nano-spotter dispenser sciFLEXARRAYER S3 (Scienion, Germany) to address tiny volumes of enzyme solution (pico- to nanoliter) at different areas on the chip surface. With both techniques, the selected enzymes will bind adsorptively to the sensor surface. The EIS sensors were modified by three different enzymes (penicillinase, urease, and glucose oxidase) by means of both different immobilization techniques. Immediately before immobilization of the respective enzyme, the sensor surface has been cleaned in acetone, isopropanol, and deionized water in an ultrasonic bath for 10 min, respectively.

**Figure 2:**
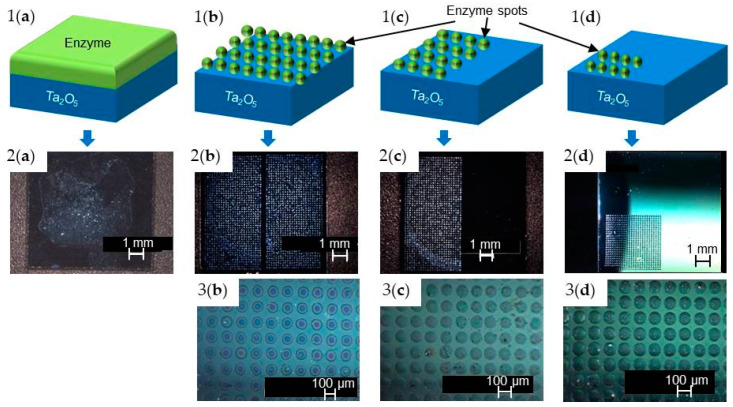
Schematic of EIS sensor modified with enzyme by means of drop-coating (sensor type 1**a**), fully spotted (sensor type 1**b**), half spotted (sensor type 1**c**) and quarterly spotted (sensor type 1**d**). Corresponding digital video microscopy images with an optical magnification of 25× (2**a**–2**b**) and 100× (3**b**–3**d**), respectively.

For penicillinase immobilization, the enzyme penicillinase was dissolved in TEA buffer solution (0.3 M, pH 6) and well stirred. An amount of 27 µL of enzyme solution was drop-coated (see Figure 2,1a) onto the EIS surface resulting in an enzyme activity of ~36 U per sensor. With the nano-spotter dispenser, 0.9 µL of penicillinase solution with a drop volume of 360 pL was fully spotted (2500 spots) onto the Ta_2_O_5_ surface with 1 cm^2^ (shown in Figure 2,1b) resulting in an equivalent activity. In this case, additional studies were performed by spotting the enzyme solution onto the sensor surface not only completely, but also half (0.5 cm^2^ of the sensor surface) with 0.9 µL of enzyme solution and 2 drops per spot (Figure 2,1c) and quarter area with 0.9 µL of enzyme solution onto 0.25 cm^2^ of the sensor surface and with 4 drops per spot (Figure 2,1d) by remaining the same activity.

As a second example, for urease immobilization, the enzyme urease was dissolved in PBS (0.33 mM, pH 6). After stirring, 50 µL of enzyme solution was drop-coated correspondingly onto the sensor surface (30 U) (Figure 2,1a). For the preparation of the EIS biosensor by the nano-spotter dispenser, 0.84 µL of urease solution was deposited with a drop volume of 336 pL onto the sensor surface, resulting in the same enzyme activity as prepared by the drop-coating technique (Figure 2,1b).

The third enzyme (glucose oxidase) was also dissolved in PBS (0.25 mM, pH 7.4). An amount of 80 µL of the thoroughly mixed solution was drop-coated onto the sensor surface resulting in an enzyme activity of ~18.5 U per sensor (Figure 2,1a). Again, for a comparison of both immobilization techniques, an enzyme stock solution was prepared to immobilize 245 pL per spot with the nano-spotter technique onto the sensor surface resulting in 0.62 µL to get the same enzyme activity (Figure 2,1b).

As the final experiment, two different enzymes (penicillinase and urease) were deposited in parallel onto two defined regions on the sensor surface by using the nano-spotter dispenser. Half of the sensor was immobilized with penicillinase (36 U) with a drop volume of 360 pL and a final penicillinase solution of 0.9 µL, and the other half with urease (30 U) with a drop volume of 366 pL and a final urease solution of 0.84 µL.

For statistical evaluation, all combinations of sensor preparations were fabricated three times each. After immobilization procedure, the EIS biosensors were mounted into a homemade measurement cell, sealed by an O-ring to define the area of the sensor chip (with ~0.5 cm^2^), which is in contact to the analyte solution.

### 2.3. Measurement Principles

In Figure 1 (left), the measurement set-up of the capacitive EIS sensor together with a commercial Ag/AgCl double-junction reference electrode (Metrohm, Germany) is shown. For electrochemical characterization of the fabricated biosensors, C/V (capacitance/voltage) and ConCap (constant-capacitance) measurements were performed by using an impedance analyzer Zennium (Zahner Elektrik, Germany) and by applying an AC (alternating current) voltage of 20 mV and a frequency of 120 Hz. To define the working capacitance in the linear range of the depletion region (~60% of the maximum capacitance), necessary for the implementation of ConCap measurements, C/V curves of each sensor chip were carried out in a DC (direct current) voltage range between –2 V and 2 V with steps of 100 mV (data not shown). For more details in measurement set-up and field-effect measurements, see [27,28].

Three different analytes have been chosen to compare the functionality of the nano-spotting immobilization technique over the conventional drop-coating technique. The measurement principle of the capacitive EIS sensor with the particularly immobilized enzyme onto the pH-sensitive transducer surface (here: Ta_2_O_5_) can be explained by the enzymatic reactions, depicted in Equations (1)–(3) [28]:

(1) $${\mathrm{Penicillin}\;+\;H}_{2}O\;\overset{\mathrm{Penicillinase}}{\to }{\;\mathrm{Penicillinoic}\;\mathrm{acid}\;+\;H}^{+}$$

(2) $${\mathrm{Urea}\;+\;3H}_{2}O\;\overset{\mathrm{Urease}}{\to }{\;2\;\mathrm{NH}}_{4}^{+}{\;+\;\mathrm{OH}}^{-}{\;+\;\mathrm{HCO}}_{3}^{-}$$

(3) $$\begin{array}{c} {\mathrm{Glucose}\;+\;H}_{2}{O\;+\;O}_{2}\;\overset{\mathrm{GOD}}{\to }{\;\mathrm{Gluconolactone}\;+\;H}_{2}O_{2}\\ {\mathrm{Gluconolactone}+H}_{2}O\;\to {\;\mathrm{Gluconic}\;\mathrm{acid}+H}^{+} \end{array}$$

The catalytical hydrolysis of penicillin by the enzyme penicillinase (Equation (1)) results in a pH decrease at the interface between enzyme and Ta_2_O_5_ layer (increase of H^+^ ions). Thus, the resulting change in the flat-band potential of the EIS sensor can be recorded. Figure 1a shows schematically the expected ConCap characteristic with increasing penicillin concentrations. The higher the penicillin concentration, the more H^+^ ions are generated due to the enzymatic catalysis in Equation (1). Ideally, one expects such typical step-like behavior, at least in the linear region of the later-on calibration plot. A similar behavior is also expected during the measurement of glucose (Equation (3), Figure 1a). Instead, during the enzymatic reaction of urea catalyzed by the enzyme urease (see Equation (2)), a pH increase, and hence a voltage increase will be expected due to the increase of OH^-^-ion concentration at the sensor surface (Figure 1b). In case of multi-enzyme immobilization via nano-spotting dispenser, depending on the analyte, the measured potential will either decrease (penicillin) or increase (urea) (see Figure 1c).

All measurements were carried out in a Faraday cage at room temperature. Before starting the measurements, the different sensors (for each type, N = 3) were incubated for 30 min in 0.25 mM PBS (at the appropriate pH value). The measurements were performed by applying 1 mL of analyte solution onto the sensor surface containing different analyte concentrations (penicillin, urea, and glucose).

## 3. Results and Discussion

### 3.1. Optical Characterization of Different Enzyme Immobilization Techniques

Immobilization of the three different enzymes (penicillinase, urease, and glucose oxidase) at a selected area on the surface of the EIS sensor with spatially resolved spots was investigated by nano-spotting (see Figure 2, sensor types (b–d)) and compared with conventional drop-coating technique (see. Figure 2, sensor type (a)). The surface of the EIS biosensors modified by the particular enzyme was characterized by 3D digital video microscopy (VHX-100 Keyence, Germany) at an optical magnification of 25× and 100×. The images presented in Figure 2,2a–2d and 3b–3d exemplarily overview the different variants of immobilization with the enzyme penicillinase. The EIS sensor surface modified by drop-coating (Figure 2,2a) depicts a completely covered sensor surface with penicillinase, where parts are shining slightly brighter, which can be explained by formation of enzyme agglomerates. In comparison to that, EIS sensors modified by enzyme nano-spotting (fully, half-, and quarter-spotted) onto the Ta_2_O_5_ layer are shown in Figure 2,2b–2d and 3b–3d. The nano-spotter dispenser yields nano-droplets of enzyme cocktail with a mean diameter of about 84 ± 9 µm when only one drop per spot was applied (fully spotted sensor surface (b) with 2500 spots per sensor). To ensure the same enzyme activity onto the sensor surface, two droplets per spot (1250 spots per sensor) containing the enzyme penicillinase were deposited resulting in a final mean diameter of about 93 ±7 µm. Four droplets per spot (625 spots per sensor) with a mean diameter of about 102 ± 9 µm were deposited to realize the quarter-spotted sensor having the same enzyme activity. For all types of sensors, spot deposition was highly reproducible and homogeneous. Similar behavior was found for immobilization of urease and glucose oxidase, respectively (data not shown). A special focus in this experiment was devoted to the modification of the sensor surface with spatially resolved enzyme spots, which could be perfectly realized in all three cases (fully, half- and quarter-spotted) at a defined area without additional patterning of the sensor surface. 

Although immobilization by nano-spotting technique enables spatial-resolved deposition of the three selected enzymes penicillinase, urease, and glucose oxidase, some limitations must be taken into account when applying this technique: (i) The spot volume for each individual enzyme must be optimized with regard to the spot geometry; independent from the specific capillary used by the manufacturer (Scenion, Germany), slight variations in the volumes (245–360 pL/drop) are necessary to obtain “ideally” rounded, circular droplet spots. (ii) The immobilization surface plays a crucial role, i.e., the more hydrophobic or hydrophilic behavior will strongly influence the spot geometry (which is, however, also the same for drop-coated immobilization). (iii) Both, the drop volume and spot-to-spot distance must be finally optimized in terms of guaranteeing to distinguish single spots properly. On the other hand, the applied nano-spotting technique refers on high reproducibility, flexibility, and reliability. As described in [29], the mechanical precision is indicated as <5 µm. The applied volume can be chosen between 10 pL and 100 µL with an average coefficient of variation of <2% per drop volume (typically 0.5%). For the localization of a spot at a certain area of the sensor surface, a live-stream camera is helpful to adjust the area of interest. According to the data sheet of the applied nano-spotter, the step size can be justified by up to 5 µm [29]. 

### 3.2. Electrochemical Characterization of EIS Biosensors with Penicillinae, Urease, and Glucose Oxidase

**Figure 3:**
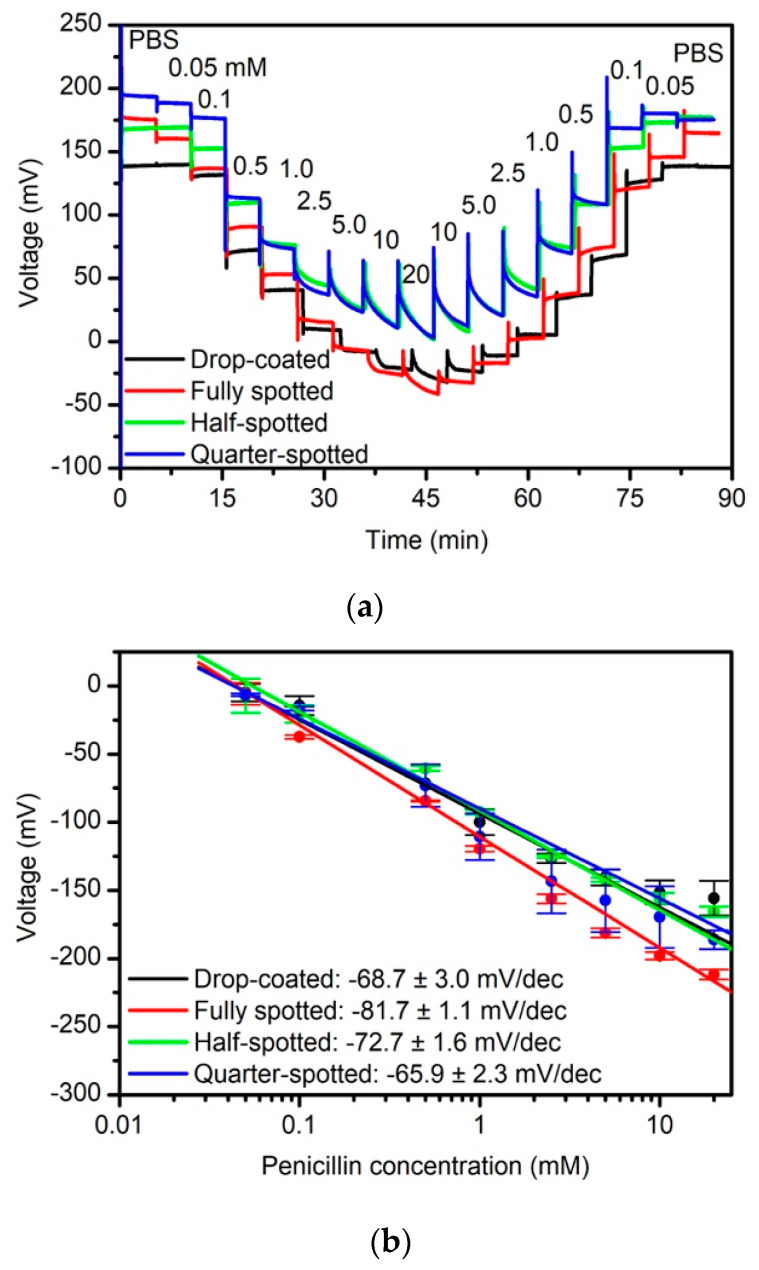
(**a**) ConCap measurements of different prepared EIS biosensors modified with the enzyme penicillinase (drop-coating: black curve, fully spotted: red curve, half-spotted: green curve and quarter-spotted: blue curve) measured in PBS, pH 8, with different penicillin concentrations from 0.05 mM to 20 mM; (**b**) corresponding calibration curves evaluated from ConCap response in the linear concentration range; PBS: polymix multi-component buffer solution; number of investigated sensors for each type N = 3.

The influence of nano-spotting immobilization onto the different enzymes penicillinase, urease, and glucose oxidase as well as the immobilization of the particular enzyme at defined areas of the sensor chip (fully, half-, quarter-spotted) have been investigated with regard to the intrinsic biosensor characteristics by means of ConCap measurements. In case of the penicillin biosensor, penicillinase concentrations varied in the range between 0.05 mM and 20 mM, dissolved in 0.25 mM PBS, pH 8. Each concentration was recorded for 5 min. Typically, the measurements started with buffer solution (PBS, pH 8), followed by the lowest concentration of penicillin (0.05 mM) and with a step-wise increase to the highest concentration of 20 mM. Then, the penicillin concentrations were step-wise reduced to a penicillin concentration of 0.05 mM. At the end, again the measurements were performed in buffer solution (PBS, pH 8) for 5 min. The measurements were carried out at room temperature in a Faraday cage. Figure 3 shows typical dynamic response curves (a) of the different capacitive EIS biosensors (drop-coating, fully, half- and quarter-spotted) and the corresponding calibration curves (b). Note, that all prepared EIS sensors were modified with the same enzyme activity of ~36 U (only by varying the spot counts and applied drop numbers per spot). Three sensors have been prepared for each sensor type for statistical reasons.

As expected, a clear dependence of the sensor signal on different penicillin concentrations can be recognized for all biosensor types. Due to the enzymatic reaction in Equation (1), H^+^ ions were released, lowering the pH value at the pH-sensitive Ta_2_O_5_ layer, and resulting in a voltage decrease. Clear signal steps independently of the upward or downward concentration series of the performed measurements can be achieved with a similar behavior for all four sensor types. The average penicillin sensitivity in the linear concentration range (0.05 mM to 20 mM) is 81.7 ± 1.1 mV/dec (fully spotted), 72.7 ± 1.6 mV/dec (half-spotted) and 65.9 ± 2.3 mV/dec (quarter-spotted). In comparison, for the drop-coated penicillin biosensor, an average penicillin sensitivity of 68.7 ± 3.0 mV/dec was found. The obtained data for penicillin sensitivity are in good agreement with conventional immobilization procedures discussed in literature [30], and thus underline the feasibility of nano-spotting technique for enzyme immobilization. Interestingly, a somewhat higher penicillin sensitivity exists in case of the fully spotted enzyme (see Figure 2b), whereas the “lowest” sensitivity remains for the quarter-spotted EIS biosensor. 

Even though, the variation in penicillin sensitivities is in the margin to be expected, this trend might be explained by two reasons: i) In case of the fully spotted immobilization, one layer of spots is deposited on the sensor surface, whereas for the half- and quarter-spotted immobilization two and four spot layers (two drops per spot/four drops per spot), respectively, may slightly hinder a free diffusion of substrate molecules (penicillin) as well as products (H^+^ ions) to the pH-sensitive transducer surface. ii) For the half- and quarter-spotted enzyme immobilization, the size of sensor-active region is correspondingly decreased, changing the ratio between enzyme-covered and enzyme-free region on the sensor surface, which might negatively influence the diffusion behavior of its substrate to be detected.

**Figure 4:**
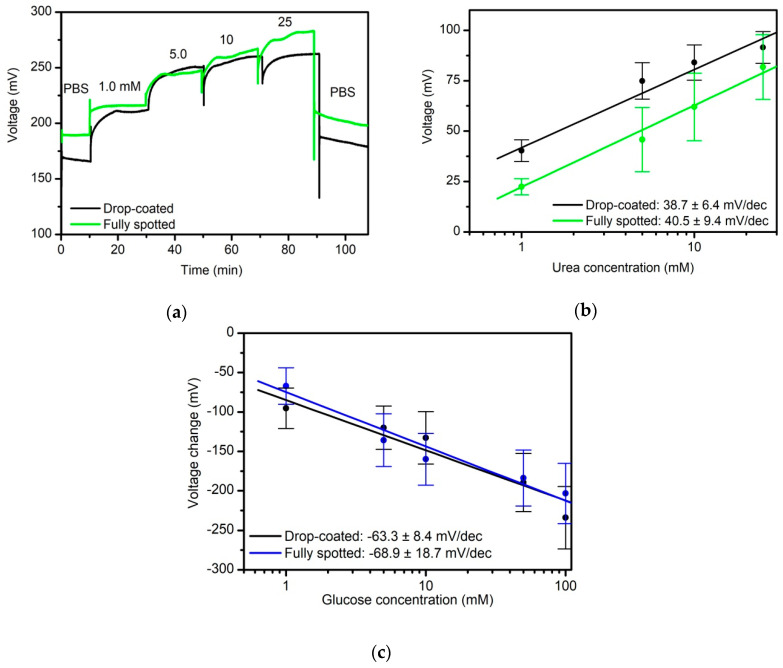
(**a**) ConCap response of EIS biosensor modified by the enzyme urease by drop-coating (black curve) and fully spotted (green curve) in PBS, pH 6.8, and urea solution of different concentrations from 1 mM to 25 mM; (**b**) corresponding calibration curves for both sensors; (**c**) calibration curves evaluated from ConCap response (data not shown) for the nano-spotted capacitive glucose oxidase-modified EIS biosensor (blue) and the drop-coated biosensor (black) recorded in PBS, pH 7.4, with different glucose concentrations from 1 mM to 100 mM.PBS: polymix multi-component buffer solution; numer of investigated sensors for each type N = 3.

Figure 4 demonstrates the applicability of the nano-spotting technique using the enzymes urease and glucose oxidase. Exemplary ConCap recordings (PBS, pH 6.8) of different urea concentrations between 1.0 mM and 25 mM urea for the drop-coating and nano-spotted EIS biosensor, respectively, are shown in Figure 4a. Each concentration was recorded for 5 min. The measurements were carried out at room temperature in a Faraday cage. At first, the measurements were performed in buffer solution (PBS, pH 6.8), followed by the lowest concentration of urea (1 mM). The urea concentrations were step-wise increased to the highest concentration of 25 mM, before performing the measurements again in buffer solution (PBS, pH 6.8) for 5 min. Additionally here, both capacitive EIS biosensors indicate a similar sensor behavior. The signal steps with varying urea concentration are clearly visible and increase with higher urea concentration, due to the enzymatic reaction in Equation (2), where the pH value shifts to more alkaline regime. The obtained sensitivities of 38.7 ± 6.4 mV/dec for the sensor modified by drop-coating technique and 40.5 ± 9.4 mV/dec for the fully nano-spotted sensor in the linear concentration range from 1.0 mM to 25 mM show the very good conformity between both immobilization procedures; see also data in [31,32,33]. At the same time, to our best knowledge, results of a capacitance EIS biosensor with nano-spotting immobilization of urease for urea detection have not been discussed yet.

The same nano-spotting/drop-coating experiment was performed with glucose oxidase as third model enzyme. Figure 4c depicts the calibration curves for varying glucose concentrations between 1 mM and 100 mM, PBS buffer, pH 7.4. Each concentration was recorded for 5 min at room temperature in a Faraday cage. At first, the measurements were performed in buffer solution (PBS, pH 7.4), followed by the lowest concentration of glucose (1 mM) and with a step-wise increase to the highest glucose concentration of 100 mM, and ending up with PBS buffer. As expected, with increasing glucose concentration, the sensor signal is also decreasing, since H^+^ ions were released to build gluconic acid, see Equation (3). The mean values of the resulting glucose sensitivities of three measurement series of three individually prepared EIS biosensors calculated in the linear range between 1 mM and 100 mM results in 68.9 ± 18.7 mV/dec (nano-spotted immobilized) and 63.3 ± 8.4 mV/dec (drop-coated), respectively. As for the penicillin and urea biosensor, comparable sensitivities were achieved, when applying drop-coating or nano-spotting immobilization. In contrast to penicillin and urea biosensors, only rare information about capacitive field-effect sensors for glucose monitoring can be found in literature. Nonetheless, the glucose sensitivity values are in good agreement with reference data [34,35].

**Figure 5:**
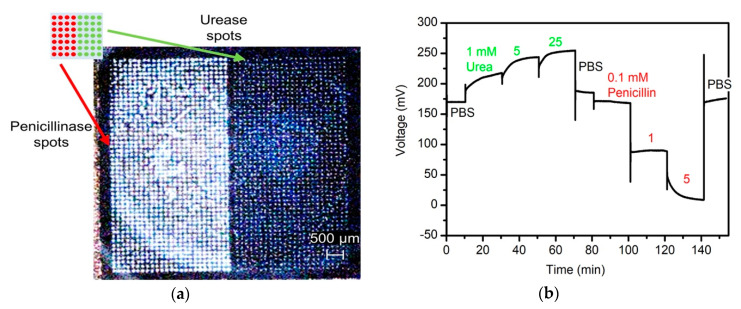
(**a**) 3D video microscopic image of sensor surface with two immobilized enzymes by nano-spotting (schematic inlet on top for more clarification). (**b**) ConCap measurement of EIS multi-enzyme biosensor modified with half-spotted urease and half-spotted penicillinase measured first in polymix buffer solution and with different urea concentrations (1 mM to 25 mM), and then in PBS buffer and different penicillin concentrations (0.1 mM to 5 mM).

The key experiment in this study was to investigate the simultaneous immobilization of several enzymes onto a capacitive field-effect sensor under controlled conditions. The multi-enzyme immobilization was performed with the enzymes urease and penicillinase via nano-spotting technique; that is, the sensor was modified using adsorptive immobilization technique. Figure 5a shows the 3D video microscopic image of the sensor chip with both immobilized enzymes (the schematic (top left) additionally explains the immobilization set-up). These two enzymes have been particularly selected due to their contrasting pH shift triggered by the respective enzymatic reaction. While the pH is increasing with increasing urea concentration catalyzed by the enzymatic reaction of urea indicated by an increase in the voltage, the voltage will decrease with higher penicillin concentration due to the reaction with the enzyme penicillinase. Exactly this behavior is shown in the ConCap graph in Figure 5b. The biosensor signal was first recorded during the measurement of PBS at room temperature in a Faraday cage. Then, different urea concentrations (1 mM to 25 mM) were spiked into the buffer solution. The corresponding potential increase is visible. Then, after measuring in PBS for a second time, penicillin in the concentration range between 0.1 mM and 5 mM was added to the buffer solution resulting in a decrease of the biosensor signal. At the end, only PBS was measured where the recorded signal value of ~176 mV is comparable with the initial starting value of ~170 mV. This capacitive EIS biosensor simultaneously modified with two enzymes (urease and penicillinase) demonstrates that multiple analyte detection with one sensor is possible. 

Usually, capacitive field-effect biosensors cannot be separated as multi-sensor arrangement, in comparison to more complex ISFET (ion-sensitive field-effect transistors) arrays, where different gate regions can be addressed individually. At the same time, ISFETs require elaborate photolithographical patterning and passivation/encapsulation, whereas EIS biosensors benefit from a flat sensor surface, which can be e.g., simply sealed by an O-ring in a home-made measurement cell [36,37]. Here, we successfully demonstrated for the first time the combination of two enzymes on the same EIS sensor surface. Note, the measurement in Figure 5 shows (i) both enzymes were working independently, having roughly the same activity as for the half-spotted sensor type used in Figure 3 and Figure 4, respectively; and (ii) when monitoring the different urea concentrations, the penicillin-sensitive part remains unaffected and vice versa.

## 4. Conclusions

A capacitive EIS field-effect biosensor was modified by two different immobilization techniques, namely conventional drop-coating and nano-spotting. Both methods were systematically compared with each other by investigating three different enzymes: penicillinase, urease, and glucose oxidase. With the nano-spotting immobilization technique, a controlled and spatially resolved immobilization (fully, half, and quarter) of different enzyme-solution volumes onto the sensor surface is possible. Results obtained with nano-spotting technique convince of high reproducibility, high grade of automation, and high flexibility in terms of tailoring sensor-active regions on the sensor surface. An average sensitivity of the sensor modified by the nano-spotter could be reached for penicillin: 81.7 (mV/dec), urea: 40.5 mV/dec, and glucose oxidase: 68.9 mV/dec. 

Further advantages of the nano-spotting over drop-coating immobilization are the less amount of valuable enzyme solution (picoliter), which can be immobilized as droplet spots with a defined size and at a selected local place. Such droplets allow having a size of ~100 µm in diameter, which makes the miniaturization of biosensors with immobilized enzyme achievable.

The main benefit of nano-spotting immobilization is the combination of several enzymes onto the same sensor surface, which has been exemplarily demonstrated by the enzyme couple urase/penicillinase. Both enzymes catalyzed their respective substrates without affecting each other.

In future studies, the main focus will be given on the immobilization of a wide variety of possible enzyme combinations onto the same sensor surface of a capacitive field-effect sensor to study the efficiency of this nano-spotting technique. We believe that due to the application of the nano-spotting immobilization technique, a new route of capacitive multi-enzyme field-effect biosensors for multi-analyte detection is launched, which can have a great potential for practical applications in e.g., medicine, environmental monitoring, or bioprocess control, to facilitate and accelerate the analysis. From the scientific point of view, further scenarios might be to either modify different areas of the sensor surface by different enzymes (such as each quarter or eighth) or to vary spot-wise the composition of enzyme cocktail for immobilization. Both directions enable multi-parameter monitoring on a single chip; however, the interaction between the enzymes of the micro-spots as well as the cross-sensitivity has to be proven, too.
